# The Zeitraffer Phenomenon: A Strategic Ischemic Infarct of the Banks of the Parieto-Occipital Sulcus - A Unique Case Report and a Side Note on the Neuroanatomy of Visual Perception

**DOI:** 10.7759/cureus.9443

**Published:** 2020-07-28

**Authors:** Hassan Kesserwani

**Affiliations:** 1 Neurology, Flowers Medical Group, Dothan, USA

**Keywords:** ischemic cva, neural sulcus, vision disturbance

## Abstract

We describe the remarkable case of a medically healthy right-handed 15-year-old boy who developed an ischemic infarct of the banks of the right parieto-occipital sulcus (POs). The etiology of this infarct was undetermined, that is, cryptogenic. However, the focus of this article is functional neuroanatomy, as our patient developed a specific entity; an optic flow motion deficit characterized by slow self-motion of the left half of his body (egomotion) and slow motion of the surroundings (allocentric motion) together constituting the Zeitraffer phenomenon. We describe the neuroanatomy and neurophysiology of the dorsal visual stream and correlate the localization of this type of infarct in our patient with the known functional neuroanatomy. Although lesional studies in Macaque monkeys and functional MRI studies in humans have documented the clinical-functional correlations of POs lesions and perceived motion deficits, our case is one of the very first human cases in the literature that pinpoints the Zeitraffer phenomenon to a specific and strategic circumscribed ischemic stroke in the region of the POs.

## Introduction

Before embarking on the trajectories of the visual processing pathways, we need to note that the cortical areas directly receiving visual information from the lateral geniculate nucleus (LGN) include the striate cortex, visual area V1 and the extrastriate cortices V2, V3, V4 and V5. Area V6 includes the dorsomedial area, the cortex along the medial longitudinal fissure and the banks of the parieto-occipital sulcus (POs).

Visual processing of information is described by the two stream hypothesis: the ventral and dorsal stream. The ventral stream or "what pathway" translates object identification and recognition. It flows from the parvocellular region of the LGN and projects to area V1 of the striate cortex. It then flows through areas V2 and V4 of the visual association cortices to the inferior temporal lobes. A lesion here can lead to prosopagnosia and lack of recognition of shapes and objects [1]. The dorsal stream or "where pathway" emerges from the striate cortex V1 and bifurcates into two steams that terminate in the parietal association cortex. The dorsolateral stream terminates in area V6 and medial intraparietal (MIP) area of the superior parietal lobule and is involved in object and self-motion recognition in the whole visual field. The dorsomedial stream ends in the middle temporal (MT)/V5 area of the inferior parietal lobule and is involved in the speed and direction of motion of the central field of vision [2]. Lesions of the dorsal visual pathways can lead to impaired visual guidance of the limbs (optic ataxia), inability to synthesize a scene (asimultagnosia), slow or inability to perceive motion (akinetopsia), hemispatial neglect or inability to count a small cluster of points spontaneously (subitization) [3].

The Zeitraffer phenomenon is the perception of altered speed of motion of an object, either slow or fast. The preponderance of right hemispheric lesions is a striking phenomenon. It has been associated with lesions of the dorsolateral stream, area V5 and its connections. It has been described with ischemic infarcts and head injuries, as a migrainous phenomenon and in epileptic seizure semiology [4-7].

We describe a 15-year-old boy without known risk factors for ischemic stroke, who developed a strategic infarct of the banks of the POs and manifested predominant slow egocentric and slow allocentric perception of motion, the Zeitraffer phenomenon. We feel this is a unique case because of the well-circumscribed lesion around the POs. This is in keeping with lesions of the dorsolateral stream, as outlined above. This further strengthens the existing data from lesional studies of Macaque monkeys (area MT) and functional MRI (fMRI) studies of humans, as we demonstrate it in an explicit fashion in vivo in a human [2].

## Case presentation

We describe the case of a 15-year-old right-handed boy, who one day after a swim at the local beach decided to take a nap. When he woke up, he noted blurring of vision of the left half of his visual field. He moved his left arm and noted it was moving in slow motion. He did not compare the left and right arm. He raced to report this phenomenon to his mom and he noted that his sister's babysitter was walking in slow motion. It was not clear if he distinguished the slow motion of his left arm from bona fide weakness. However, his mom noted that he was clumsy while grasping a cup with his left hand, potentially an optic ataxia. He complained of similar symptoms to his left leg, yet again we were unable to ascertain definite clumsiness, in addition to slow motion, the latter being definite. He noted fleeting and transient paresthesias of his left arm and leg. His speech remained fluent but his mental acuity was blunted. He was an avid reader with an eidetic memory for languages and culture, a bona fide savant. But his mother had noted that he was unable to grasp information magnetically, as he did before. He remained remarkably calm during this whole ordeal. By 12 hours, the visual blurring and slow motion perception had resolved.

His past medical history was significant for autism spectrum disorder and obsessive compulsive disorder. It was also noted that his symptoms, specifically the blurring of vision, were magnified with the intrusion of obsessive thoughts. His family history was negative for premature coronary artery disease or cerebrovascular disease. His father had a history of deep venous thrombosis. His medications included aripiprazole, pimozide, asenapine and metaformin. The metformin was included as an antidote to the diabetic inducing properties of these three atypical neuroleptics. The pediatric psychiatrist deemed it absolutely necessary for the patient to remain on three dopamine antagonists in order to control his obsessive thoughts and behavior. After a long struggle at school, this highly intelligent young man was schooled at home.

On examination, his height was six feet (93rd centile), his weight was 172 pounds (92nd centile), BMI of 23.3 (82nd centile), BP of 115/75 mmHg, temperature 95.5 degrees Fahrenheit and pulse of 110. Precordial examination with a stethoscope did not reveal a cardiac murmur, and there was absent bruits upon auscultation of the skull and carotid arteries. His gait was perfectly normal, and he could easily tandem and stand effortlessly on either leg. His mental state was sound. He was a little withdrawn, not his usual inquisitive and talkative nature. He seemed a little distracted but answered questions appropriately. His two-minute delayed memory was one out of three words. His digit span was five out of seven. Visuomotor skills were preserved with pantomime. No limb kinetic apraxia or ideomotor apraxia was noted bilaterally with coin deftness and transitive actions, respectively. His speech was fluent, naming intact and understanding effortless. No dysarthria was noted. Pertinent cranial nerve examination revealed full ocular motion with preserved accommodation and absent ptosis. Visual fields were full to confrontation and visual field neglect absent with sequential unilateral and bilateral visual field confrontation. No facial weakness was noted. His tongue protruded to the midline.

Motor examination using Medical Research Council (MRC) scale was 5/5 throughout all the muscles of the upper and lower limbs, with one exception; sequence motion of the fingers of the left hand was subtly clumsy, and finger spreaders of the left hand were graded at 4/5. His reflexes were lively throughout the upper and lower extremities. Pertinent sensory examination revealed normal stereognosis and graphesthesia of the left hand, with intact two-point discrimination of the left index finger. Touch, pressure, pin prick and kinesthesia were normal in the index fingers of both hands. Cerebellar examination revealed normal alternating hand motion and the absence of dysmetria or intention tremor of the arms.

**Figure 1:**
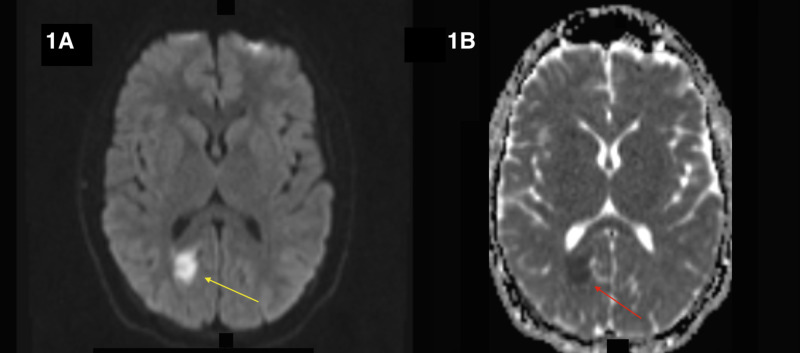
(A) Diffusion-weighted imaging (DWI) shows hyperintensity of banks of parieto-occipital sulcus (yellow arrow). (B) Matching hypointensity on gradient echo (GRE) imaging (red arrow).

Diffusion-weighted MRI revealed hyperintensity of the banks of the right POs. The ischemic nature of this lesion was confirmed by a matching hypointensity on gradient echo (GRE) imaging (Figure 1).

**Figure 2:**
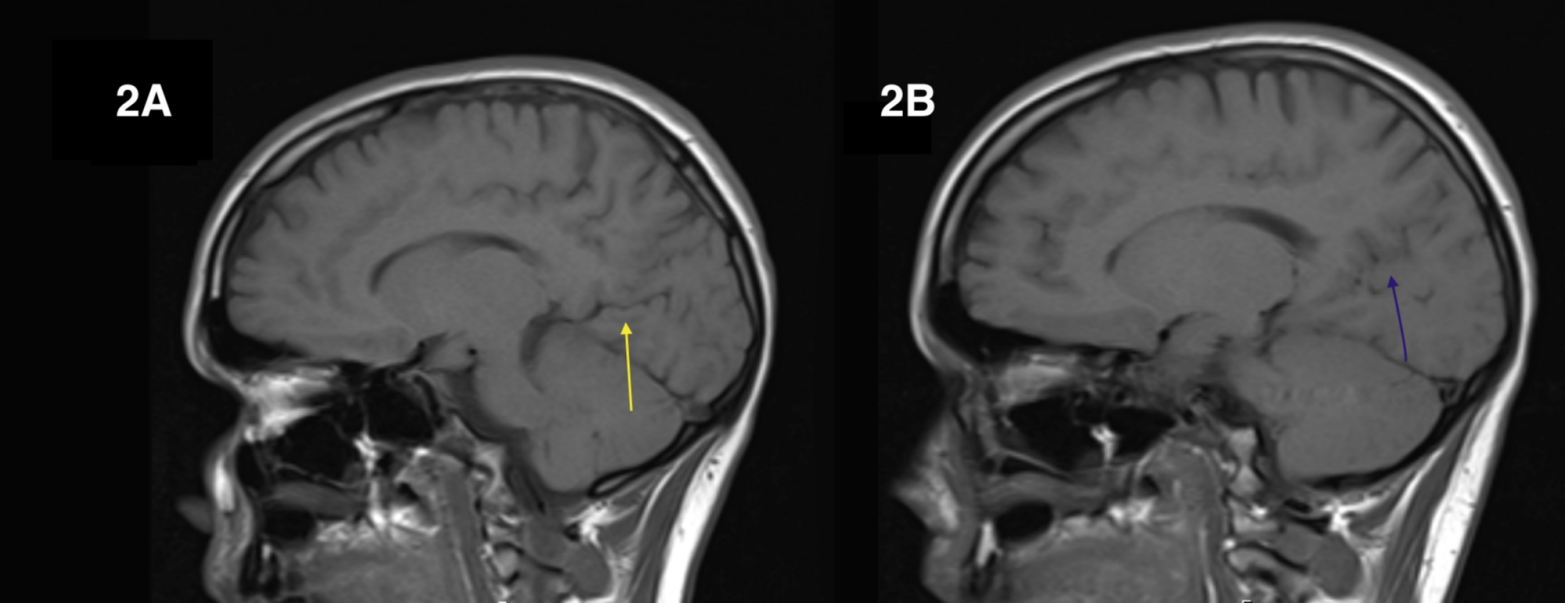
(A) A relatively linear left parieto-occipital sulcus (yellow arrow). (B) An obscured and serpiginous right parieto-occipital sulcus secondary to ischemic infarct (blue arrow).

To confirm the location of the infarct on the right POs, we compared it to the left POs, on sagittal T1-weighted images (Figure 2).

**Figure 3:**
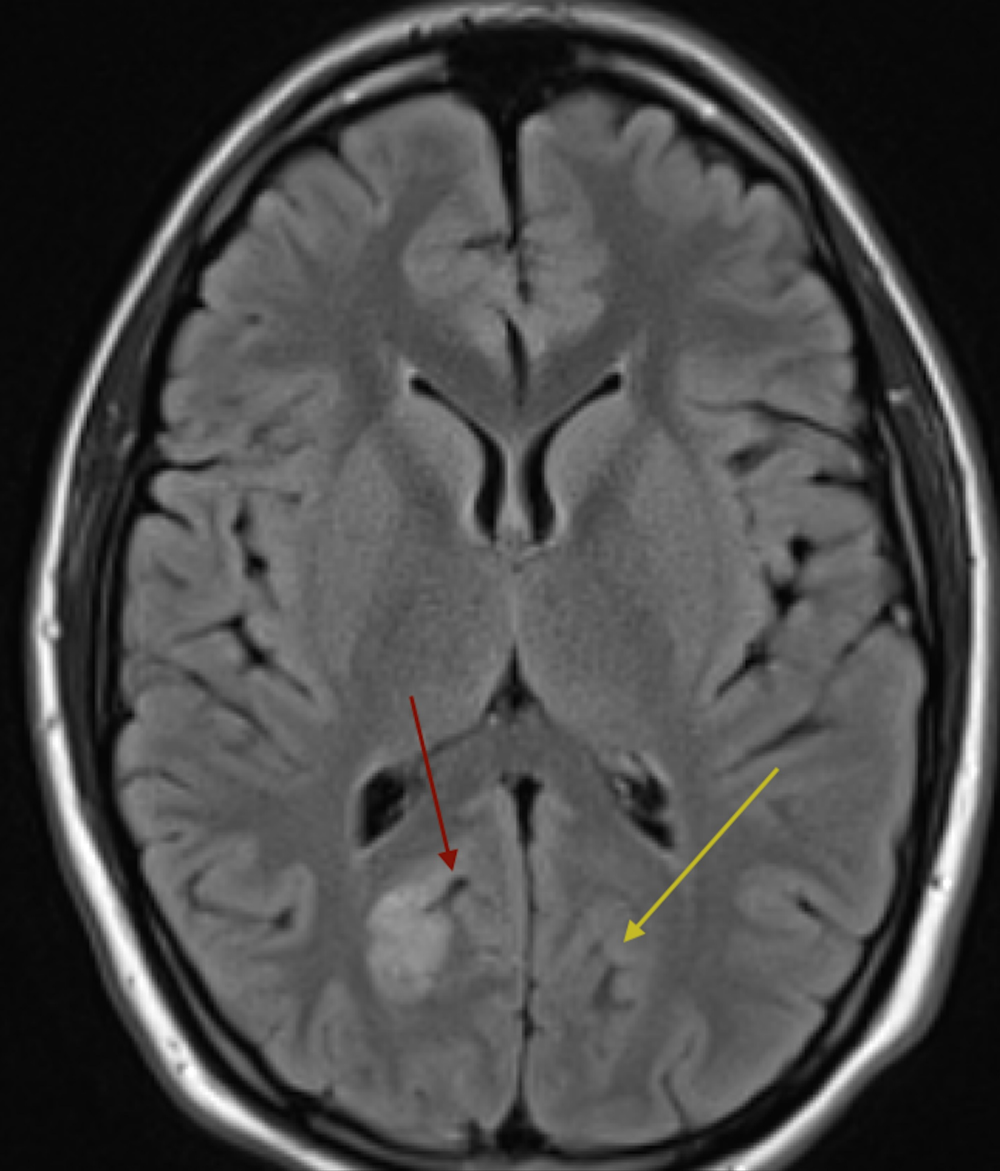
Axial FLAIR MRI demonstrates ischemic infarct obscuring right parieto-occipital sulcus (red arrow) and conspicuous left parieto-occipital sulcus (yellow arrow). FLAIR, fluid-attenuated inversion recovery

The fluid-attenuated inversion recovery (FLAIR) MRI image confirms further that the ischemic infarct is in the vicinity of the POs (Figure 3).

A CT angiogram of the brain and carotid duplex scan were normal. The usual embolic infarct workup was carried out. The patient was telemetry monitored, and a transthoracic echocardiogram showed no evidence of valvular vegetations, cavitary thrombus or atrial septal defect. The pediatric cardiologist deemed that a transthoracic echocardiogram was adequate interrogation of the heart for a 15-year-old boy. A lumbar puncture revealed a non-inflammatory cerebrospinal fluid. A full thrombophilia panel included protein C, S, anti-thrombin III activity, prothrombin G20210 A mutation, IgG and IgM antiphospholipid antibodies, beta-2-glycoprotein and lupus anticoagulant, all of which were entirely normal, except for a factor V Leiden mutation. Fasting blood lipids, including cholesterol, low-density lipoprotein (LDL) cholesterol, high-density lipoprotein (HDL) cholesterol and triglycerides, were normal. Lipoprotein A was elevated at 99 (<75 nmol/l). Two covid-19 polymerase chain reaction (PCR) tests of nasal swabs were negative on two separate occasions, five days apart. Based upon these findings, a diagnosis of a cryptogenic ischemic infarct was made. 

Although an ischemic infarct in a 15-year-old boy is significant enough to warrant an exhaustive evaluation for a specific etiology and may in itself constitute a case report, this is not the focus of this article. This case report highlights the phenomenon of Zeitraffer and localizes this phenomenon for the first time to a circumscribed lesion of the banks of the POs, which is in keeping with the literature, specifically an interruption of the dorsolateral visual stream. In our case report, we have a specific strategically localized lesion along this pathway. Other reports emphasize lesions of V6, MIP, the superior parietal lobule and en mass lesion of the whole area [2].

## Discussion

Our case localizes the phenomenon of Zeitraffer, distorted perception of speed of motion of self or objects or both, to a specific lesion of the POs along the dorsolateral stream visual pathway that extends anywhere from V1 along the banks of the POs, and terminates on area V6 of the superior parietal lobule, the association cortices. The bifurcation of the visual pathways into dual parallel processing streams has been known since 1992 [8]. These are labeled as the parvocellular (P) and magnocellular (M) pathways. The P pathway starts at the retinal midget ganglion cells and projects to the LGN of the thalamus. The P pathway then projects to a subregion of layer 4c of the striate cortex V1, which then projects to the association cortices V2 and V4. The P pathway subserves fine detail including color information and high-resolution spatial frequency. However, the M pathway begins at the larger parasol retinal ganglion cells, synapses at the magnocellular layers of the LGN of the thalamus and then projects to the striate cortex subregion of V1. The M pathway, in turn, bifurcates into two paths: the dorsomedial stream that projects to MT/V5 of the inferior parietal lobule, and henceforth to the middle superior temporal (MST) association cortex. The other is the dorsolateral stream that relays to areas V6 and MIP in the superior parietal lobule. The M pathway subserves so called "coarse" features, such as direction and speed (Table 1).

**Table 1:** Comparison of parvocellular (P) and magnocellular (M) pathways: medial intraparietal (MIP) and middle temporal (MT)

P Pathway	M Pathway
What information - object recognition and identification	Where information - object location, direction, speed
Ventral	Dorsal
Begins at midget ganglion cells	Begins at parasol ganglion cells
One route	Two routes
Ends at V4	Ends at MT/V5 and V6/MIP
Subserves fine discrimination and color	Subserves speed and direction
Lesion leads to prosopagnosia and impaired object identification and recognition	Lesion leads to Zeitraffer phenomenon, optic ataxia, asimultagnosia, akinetopsia, impaired subitization

**Figure 4:**
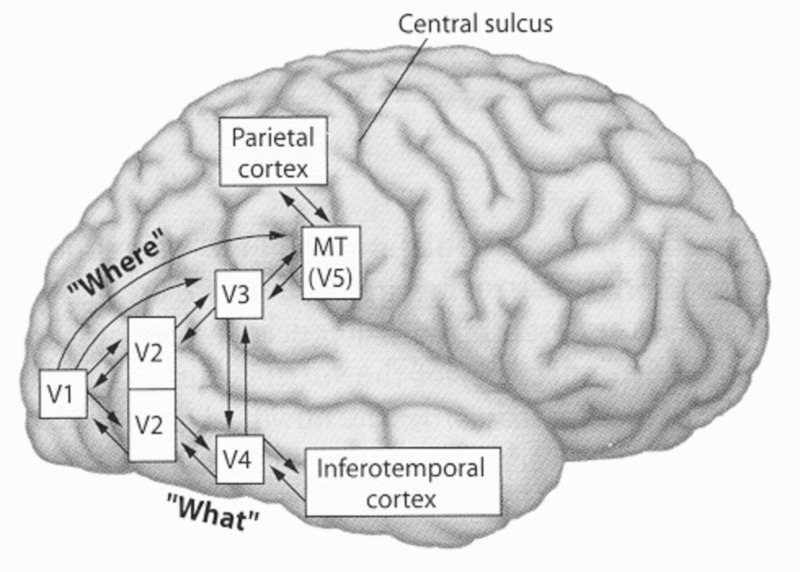
Display of ventral and dorsal streams (truncated version): middle temporal (MT).

These pathways are displayed in Figure 4.

These pathways are not mutually exclusive. There is cross communication via the vertical occipital fasciculus (VOF). These fibers course between areas V3A/B and the posterior fusiform gyrus [8]. The POs delimits the occipital lobe from the parietal lobe. It separates the precuneus (parietal) from the cuneus (occipital lobe). It is mostly visible on the medial surface, running diagonally and meeting the calcarine sulcus, behind the splenium of the corpus callosum. It runs a short course on the lateral surface, about 5 cm anterior to the occipital pole, for about 1.25 cm. The ventral bank, V6, is a purely visual area and the dorsal bank, V6a, is involved in reaching movements [9]. The pathways from V1 to MT/V5 and V6/MIP pass through the banks of the POs and antero-medial cuneus [10]. Any lesion along this pathway can potentially lead to the Zeitraffer phenomenon. 

However, association does not imply causation. It is conceivable that our patient's Zeitraffer phenomenon could be related to diaschisis to a connecting area. Diaschisis is a well-known phenomenon equivalent to functional deafferentation. Upstream and downstream regions are rendered electrically silent. However, our documented lesion is strategically located along the trajectory of the dorsolateral stream, which is known to transduce object speed and direction. The dorsal visual stream has been studied with fMRI studies on humans and single cell recordings in Macaque monkeys [2]. It has also been described with larger ischemic lesions of the right occipital lobe [4]. What we have demonstrated here is a well-circumscribed lesion along this trajectory. This finding would have been fortified even further with tensor MRI and tractography with a connectome map. Unfortunately, this imaging modality is only available at a few centers. We believe that the Zeitraffer phenomenon is more common, but obscured by the fact that ischemic strokes of the occipital lobes lead to hemianopia, which masks the phenomenon. Our case was unmasked by the restricted nature of the lesion.

## Conclusions

Zeitraffer is a fascinating phenomenon that has philosophical underpinnings. It involves perception of speed and indirectly time. According to Immanuel Kant, the great German philosopher, time and space encode consciousness. This is not a far-fetched concept. The epistemology of consciousness is elusive. But with the currently evolving methodologies of tensor imaging and the connectome, the complexity of this edifice of knowledge is slowly unfolding. Our current article reinforces prevailing concepts, and we demonstrate a specific lesion of the circuitry that encodes the perception of speed. This is an exercise in anatomical localization and functional correlation. And we believe that this case report is one of the very first reports of such a circumscribed strategic lesion in a human that is associated with the phenomenon of Zeitraffer. This is an area that is fertile ground for further research. 
